# Mechanism and Internal Stability of Supportive Stone Constructions

**DOI:** 10.3390/ma15093175

**Published:** 2022-04-27

**Authors:** Klaus Voit, Johannes Hron, Gerhard Frei, Renata Adamcova, Oliver Zeman

**Affiliations:** 1 Institute of Applied Geology, University of Natural Resources and Life Sciences, Peter Jordan-Street 82, 1190 Vienna, Austria; klaus.voit@boku.ac.at; 2 Institute of Structural Engineering, University of Natural Resources and Life Sciences, Peter Jordan-Street 82, 1190 Vienna, Austria; oliver.zeman@boku.ac.at; 3 Frei ZT, Civil Engineer, Consulting Engineer for Civil Engineering, 1190 Vienna, Austria; g.frei@zt-frei.at; 4 Faculty of Natural Sciences, Comenius University in Bratislava, Ilkovicova 6, 842 15 Bratislava, Slovakia; renata.adamcova@uniba.sk

**Keywords:** pitched slopes, stone pitching, supportive stone construction, concrete mortar, shear tests, rock-to-rock interface, rock-to-mortar interface, adhesion, friction angle

## Abstract

Natural stone constructions for the protection of slopes, banks and riverbeds are widely used in infrastructure engineering. These structures are made of stacked natural stones, which can be placed loosely on top of each other. Additionally, their bond behavior can be improved by using concrete mortar to fill the joints between the stones. Although such structures are now widely used, there is still a need for research concerning their inner stability and the structural design of such protective stone structures. In this study, experimentally, investigations were made to determine the force transmission and the interaction between rock and concrete mortar by deriving characteristic values of the adhesion strength and friction angle at different scales. A method for the determination of shear parameters from direct shear testing is used, considering the interaction between vertical and horizontal forces in the joint. In the course of these investigations, the roughness of the rock surface was recorded using conventional visual methods using the joint roughness coefficient (JRC) as well as via laser imaging. By applying laser scanning, a theoretical roughness factor could be derived. Furthermore, the properties of the rocks of the concrete mortar (fresh and hardened concrete mortar properties as well as a durability characteristic) were investigated in detail. It could be shown that different types of concrete mortar result in different bond strengths—expressed as tensile and shear strengths—when applied to a stone surface. The roughness of the stone surface has a positive influence on the tensile and shear strength between the stone and the mortar. Based on the test results, a failure description based on the Mohr–Coulomb fracture criterion could be determined, which can be used to calculate characteristic parameters for the design of stone support bodies. It was also shown that the stone’s compressive strength is being exceeded through load due to very punctual contact areas. Moreover, concrete mortar differs significantly from conventional concrete in terms of its mechanical properties due to the on-site installation conditions, which allow no dynamic compaction.

## 1. Introduction

### 1.1. General

In civil engineering projects for road and railroad construction, stone structures are widely used and indispensable as a near-natural, sustainable and climate-friendly protective construction for slope and embankment stabilization made by piling stones [1]. Natural stone structures have a low environmental impact, with low energy consumption, with climate-efficient use of materials by minimizing the use of concrete and using locally available stone materials [2,3,4]. The advantages of this simple construction method were already recognized during human settlement in the Stone Age. Stone stacking was further developed and perfected by the builders of the ancient Roman Empire with the conception of different types of non-mortared and mortared masonry. At least since this time, natural stone masonry has been an indispensable part of the building culture in Europe [1].

Two operating principles of stone structures can be distinguished: support structures with an active supporting action and stone pitching, working solely by its weight. In addition, two construction types can be distinguished: non-mortared (“dry”) and mortared stone constructions, depending on whether concrete mortar is used to fill the joints, which increases the internal stability of the structures [5,6]. Friction forces are transmitted between the rocks, or between rock and mortar, and can counteract the earth pressure. In the case of a dry installation of the natural stones, the transmission of forces takes place by means of friction through point-to-point contact between the stones [7]. In mortared constructions, the individual stones are laid in a mortar bed, which increases the contact zone or contact area. This leads to an improvement in the force transmission between the stones. Cohesive forces also act within the mortar bed as well as between the mortar and the stone (adhesive strength) in addition to the friction. This leads to a reduction in structural deformations and an increase in internal stability [8].

In contrast to reinforced concrete structures, stone structures are clearly heterogeneous, anisotropic structures [1]. These are composed of individual bodies (stones), which has the advantage that these structures can tolerate comparatively high deformation rates while maintaining their load-bearing capacity, particularly in the case of the non-mortared constructions; however, the non-mortared construction method leads to a more concentrated force transmission between the single stones and to a lower erosion protection. Detailed information on construction types and design approaches can be found in [8].

In addition to the requirements for the concrete mortar, there are also requirements for the aggregates, e.g., [9,10] in such a way that sufficient strength and durability can be ensured. In mortar construction, the properties of the aggregate (strength and resistance resp.) far exceed those of concrete mortar.

The main motivation for this paper was the fact that there are only a few general approaches to the structural design and stability of natural stone structures available. Specific methods for the analysis of the shear parameters and the calculation of the inner stability of such structures are missing. Sliding and toppling in the horizontal joint, as well as mechanical rock failure, plays an essential role in the load-bearing capacity. The properties and the structural behavior of the concrete mortar in terms of strength and durability are important, as well.

### 1.2. Manufacturing Methods and Construction Types

**Figure 1:**
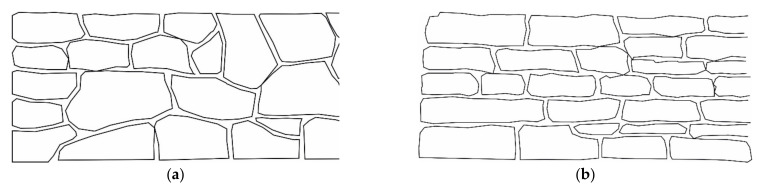
Front view of piled (**a**) and stacked (**b**) stone pitching types.

Stone constructions can be built with varying efforts and the use of equipment [8]. In the simplest case, the stones are unloaded from a truck loading area at the intended location without being moved again (a slight adjustment is still possible). The armor stones can also be unloaded near the intended place of installation. Then, the installation is conducted by a digger, placing the stones one by one or in several pieces at the desired place of installation. The greatest effort is needed when the stones are precisely placed, keeping the amount of jointing in the wall as low as possible. Each stone is selected and laid individually during the construction and turned and pressed until it fits perfectly into the structure (Figure 1). Thus, a regular and force-fit connection interface between the stones is achieved.

If the shape of the stone is irregular, it is referred to as a piled stone support structure (Figure 1a). If the stones are processed and smoothed on one or both sides, it is called stacked stone pitching (Figure 1b). As the degree of processing of the natural stones increases, the joint widths generally decrease, and horizontal joint patterns are formed [10].

Regardless of the manufacturing method, the adhesive bond in the joint can be added by using concrete mortar (Section 2.3). On the other hand, the use of grout to smooth the surface of the wall has no static effect and serves only to seal the surface [5].

Three different types of masonry can be distinguished among the construction types of stone retaining walls [1]:

- Retaining walls are mostly used in a slope cut as slope stabilization. The retaining wall, therefore, comes into contact with the ground at the foundation base, as well as at the rear wall;
- Revetment walls differ from retaining walls in that the soil behind the wall can, in principle, be considered stable; therefore, their primary purpose is to protect against erosion and weathering;
- Freestanding walls touch the ground only at their foundation base and are mostly used as a pasture or property boundary.

**Figure 2:**
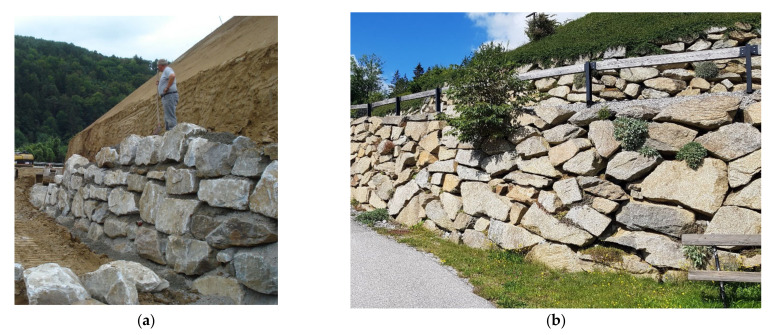
Manufacture of a piled mortared stone support body (**a**) and a piled supportive stone construction (**b**).

A retaining wall acts as a gravity wall and counteracts the acting forces from the slope behind it with the dead weight of the individual stones. The use of concrete mortar in the joints can have a favorable influence on the load-bearing behavior of the stone pitching since the contact areas are increased and there is a better force transmission between the stones. Figure 2 illustrates examples of manufacturing a mortared supportive stone construction and a non-mortared piled stone pitching some years after construction.

In this paper, only piled mortared supportive rock structures were discussed.

### 1.3. Construction Material and Interaction

The installation of natural stones and earth-dry concrete mortar (i.e., concrete of stiff consistency with a water:cement ratio ≤ 0.4) is carried out alternately, starting with the installation of the largest stones at the base (Figure 2).

Armor stones are unworked natural stones (quarry stones) obtained by blasting and/or sorting in quarries. The unworked stones are bounded by fracture surfaces or natural separation surfaces (e.g., bedding, foliation or fissure surfaces) and can be installed without significant machining [11]. The requirements for armor stone for embankments are regulated by standards [9,11] and refer to geometric requirements such as stone size, weight and shape, as well as to physical and chemical requirements to ensure sufficient resistance of the respective type of stone (e.g., limestone or granite) to environmental impacts (e.g., frost or salt crystallization).

The use of concrete mortar in the joints can have a favorable effect on the load-bearing behavior of the stone pitching since the contact areas are increased and there is a better force transmission between the stones. In addition, the shear strength of the masonry is increased by the bond strength between the stone and concrete mortar; however, mortaring can also have a negative effect: depending on the permeability of the concrete mortar layers or the bond achieved, water pressure can build up behind the stone structure in case of water ingress. The ecological function of a stone support body as a habitat for animals is also negatively affected by the use of concrete mortar [8].

In contrast to the stones, there are no normative specifications for the concrete mortar installed between the rows of stones. The use of different types of concrete is mainly based on experience regarding the functionality in relation to the environmental impact (Table 1). The composition, fresh concrete properties and the designation of the concrete mortar correspond to those of common concrete standards, e.g., [12]. The stones must be placed in the concrete mortar bed within the processing time of the concrete; therefore, concrete with extended working time is used.

## 2. Materials and Methods

### 2.1. Stone Material

Various types of rock can be used for the construction of protective stone structures; however, these must comply with the requirements of [9] to ensure adequate strength and durability. Depending on the place of usage and the corresponding geological conditions, different types of rock are used for their construction. Typical rock types are granite, gneiss, dolomite or limestone. In Austria, limestone is very often used due to its high availability along the limestone Alps; therefore, limestone of different origins was used for the tests to create test conditions as close to practice as possible. The stone usually comes from the quarry where it is freshly excavated. Accordingly, it is unweathered. The degree of fragmentation depends on the tectonic preloading of the extraction site and is usually very low as mainly compact and undisturbed rock meets the requirements of [9]. For the present investigation, drill cores from two types of limestone (Arlberg limestone and Schratten limestone, both from the Northern Calcareous Alps in western Austria) with a diameter of approximately 100 mm from exploratory drilling were used as the rock base. The testing of the contact area was carried out on large stones with a mass of 1700–2100 kg. Shear tests at the mortar-to-stone interface were conducted at a model scale of about 1:5 to evaluate the shear parameters for mortared stone support structures.

### 2.2. Concrete Mortar

#### 2.2.1. General

Compaction (use of a concrete vibrator to dynamically compact the concrete) and curing (e.g., protection of the concrete against drying out) prescribed in [12,13] for normative are not applied in supportive stone constructions. Compaction is only possible by pressing and rotating the seated stone and by poking in the joint area (static compaction), leading to more air voids and a lower concrete bulk density. This finally results in concrete mortar having different properties in comparison to standard concrete. These specific properties were investigated during this research work and are presented below.

#### 2.2.2. Concrete Mortar Characterization

Due to changes in the processing, the properties of the concrete mortar are expected to be different from normal concrete in terms of strength and durability, see also [14]. For this reason, concrete mortar test specimens were produced in the laboratory on one side and samples were taken from an existing stone support body by means of core drilling (see Section 2.2.4) on the other side to compare them. The following parameters are to be investigated: the bulk density ρ of hardened concrete, the compressive strength of cubes f_c,cube_ and the splitting tensile strength from cylinders f_t,cyl_. Table 1 shows the concrete formulations for which the characterization of the concrete properties in the laboratory was carried out.

#### 2.2.3. Durability: Frost and Freeze–Thaw Resistance

In addition to adequate strength, the durability of the concrete mortar has a significant influence regarding on the service life of supportive stone bodies. In this respect, the frost resistance and freeze–thaw resistance, in particular, play a decisive role. According to [12], the concrete composition (max. W/B value, binder content, air content) is adjusted to the respective exposure class and is accordingly achieved accordingly with the standard (dynamical) compaction. This is not the case when using static and, therefore, non-standard compaction and must be verified separately.

**Figure 3:**
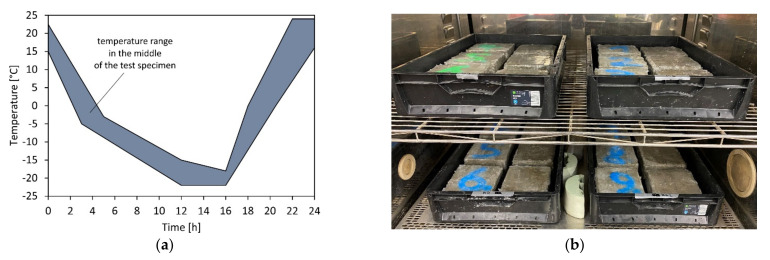
Temperature curve for the freeze–thaw tests in accordance with the specifications provided in [15] (**a**) and test performance in the climate chamber ((**b**); model Weiss WK1000).

The frost and freeze–thaw resistances were tested in accordance with [15] for concrete formulations according to Table 1 with a porous and open structure. The mass or volume of the weathering in relation to the test area was assessed after repeated freeze–thaw cycles (28 freeze–thaw cycles at +20 °C to −20 °C) using a test liquid. Water was used for determining the frost resistance, while 3% NaCl solution was used to determine the freeze–thaw resistance (test criterion for frost resistance: max. weathering of 150 cm^3^/m^2^, for freeze–thaw resistance: 250 cm^3^/m^2^, whereby the weathering between the 14th and 28th freeze–thaw cycles must be lower than between the 7th and 14th cycle). The test specimen is positioned on triangular bars and the rough-sawn test surface of a halved concrete cube (with 150 mm side length) is immersed 1 mm in the test liquid; the remaining test specimen surface is sealed impermeably to water (Figure 3) [14]. The frosting was carried out according to the temperature curve in Figure 3.

#### 2.2.4. In Situ Sampling

**Figure 4:**
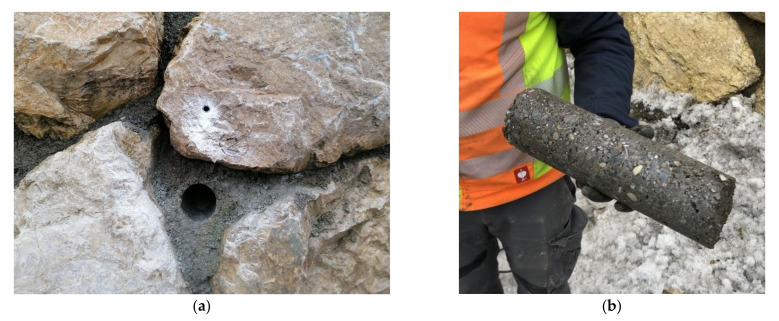
Drill core sampling from the existing supportive stone body (**a**) and extracted drill core for laboratory testing (**b**).

Drill cores were taken from mortar in a joint of an existing supportive stone body (Figure 4). During its manufacture in 2020, a concrete of strength class C25/30 and of exposure classes XC3/XD2/XF3/XA1L was used. The drill cores already show major visual differences; some are very compact (such as compacted normal concrete) and others are very open pored in some sections, which is finally reflected in the large scatter of the test results, see Section 3.1. The specimens were cut at a ratio of specimen length to the diameter of 2 to 1 to perform the compression tests. For testing the splitting tensile strength, shorter drill cores were used. Finally, the bulk density ρ of hardened concrete, the concrete compressive strength f_c,cyl_ and concrete splitting tensile strength f_t,cyl_ were determined according to [16,17,18].

### 2.3. Determination of the Contact Surface

#### 2.3.1. Non-Mortared Stone Structures

The aim was to obtain quantitative data on the extent of the contact, which is of high importance for the compressive strength. The area of the stone contact is mainly influenced by the following factors:

- Type of rock: geometry and surface (shape, sphericity and roundness) are influenced by the fracture behavior;
- Size class: the size of the rocks influences the contact zone;
- Quality of the stones: the contact area varies in size depending on the uniaxial compressive strength and stress.

**Figure 5:**
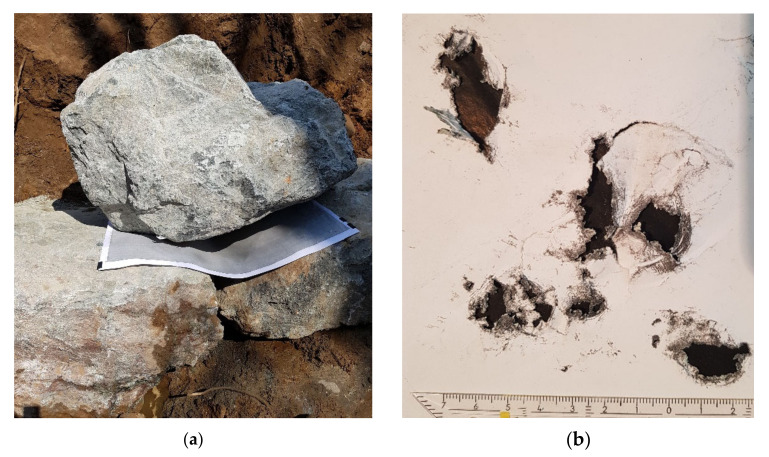
Piled stones to investigate the contact area using graphite paper (**a**) and prints of the contact areas, scale bar in cm (**b**).

Graphite paper was inserted between the test stones, which enabled us to map the contact areas digitally using CAD after scanning the graphite paper prints (black coloring in the pressure point area). Experiments were conducted both in the laboratory as well as in situ on-site during the installation of the stone construction (Figure 5).

#### 2.3.2. Mortared Stone Structures

In the case of the mortared supportive stone structures, concrete mortar is inserted after a row of stones is completed (see Figure 2), filling the joints and increasing the contact area in such a way that the stones rest on the full basal surface. Irregularities in the stone form negative shapes in the concrete surface and provide interlocking. Together with the adhesion of the concrete to the stone, this results in a bond between the individual stones and thus increases the internal stability of the stone structure.

### 2.4. Shear and Tensile Strength Testing

#### 2.4.1. Small-Scale Tests on Mortared Drill Cores

**Figure 6:**
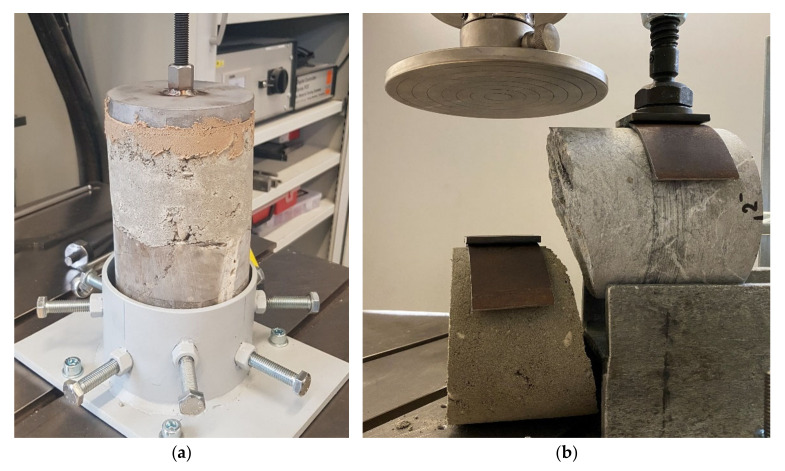
Determination of the adhesive tensile strength (**a**) and adhesive shear strength (**b**) after failure on mortared limestone drill cores.

To evaluate the adhesive shear strength and adhesive tensile strength of the stone–concrete mortar contact surface, small-scale tests were carried out with drilling cores from two different limestone types to which concrete mortar was applied. The concrete types listed in Table 1 were used. The adhesive tensile strength was determined by means of a direct tensile test derived from asphalt testing [19]. The shear test was carried out by shearing off the mortared concrete by a method also derived from asphalt testing [20] (Figure 6).

To produce the specimens, a cylindrical mold was arranged around a drill core; the concrete mortar was poured into the mold on the top of the rock and statically compacted with a ram. Sample preparation was carried out in wet and dry conditions on the surface before installing the concrete mortar. The test was carried out in a displacement-controlled testing machine (LFV 63 HH from Walter + bai ag) after a curing period of 28 days; the storage was at a temperature of 20 °C in a normal atmosphere. In addition, the surface roughness of the rock surface was measured, see Section 2.4.2.

#### 2.4.2. Laboratory Tests on Mortared Supportive Stone Structures

**Figure 7:**
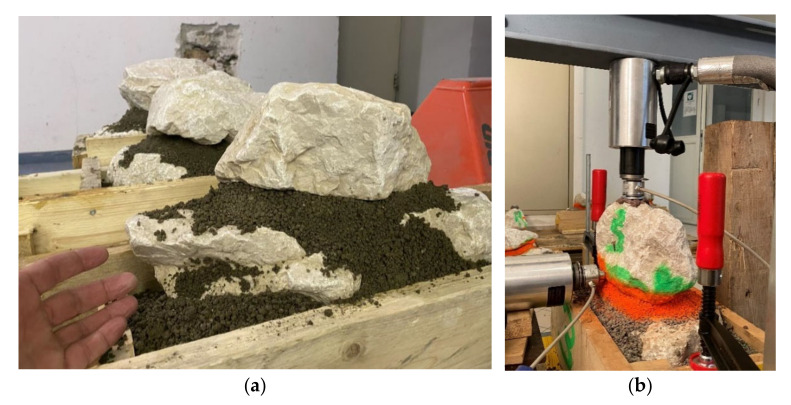
Installing stones for model tests (**a**), application of a constant vertical load and increasing the horizontal force until the failure of the structure (**b**).

To derive the shear strength parameters friction angle and cohesion (which actually defines the adhesion between mortar and stone), shear tests were carried out on mortared stones in large-scale tentative tests, as well as at a model scale of approximately 1:5 (rock dimension of approximately 30 × 20 cm^2^; Figure 7). This paper presents the experiments and results of the model tests.

To determine a fracture criterion in accordance with the Mohr–Coulomb theory via shear stress τ and compressive stress σ, direct shear tests were carried out with a constant centric load of 0 kN, 1.5 kN and 3 kN on the supportive stone body by means of a hydraulic cylinder press (Figure 7). A calotte was glued to the stone to ensure centric force transmission. The corresponding horizontal force was recorded during the loading tests until reaching the breaking load. The forces determined by load cells were converted into a stress after the test by a detailed evaluation of the contact area.

### 2.5. Determination of Surface Roughness

**Figure 8:**
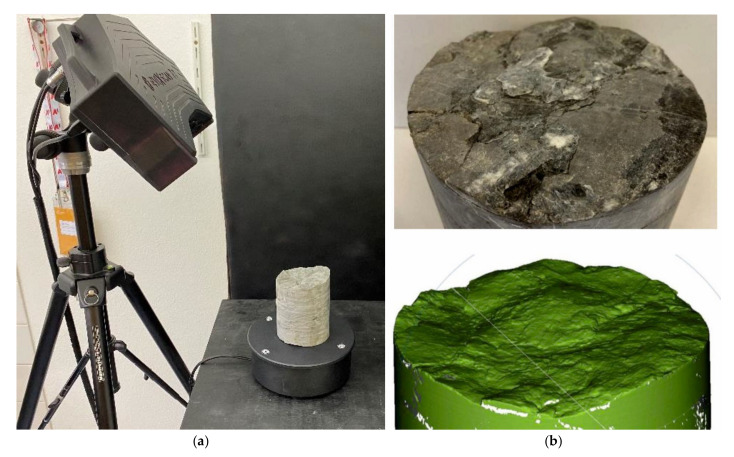
Scanning of the stone surface to determine the roughness: test configuration with the scanner (**a**), stone surface and resulting scanner image (**b**).

As far as the transmission of the forces between the stone surface and the mortar is concerned, the stone roughness plays an important role. During the production of the specimens, the surface roughness of the stone surface, on which the concrete was cast was measured visually using the joint roughness coefficient (JRC, using ten gradations, from 1 “very plain” to 10 “very rough”, see [21]). The surfaces of 20 specimens were additionally scanned with an eviXscan 3D Heavy Duty Quadro, Evatronix S.A for verification and comparison (Figure 8).

From the scanner image, the actual rock surface S_a_ could be determined by means of the scanner software. It was compared with the theoretical circular basal surface of the sample (A = π·r^2^) for the calculation of a roughness factor f_r_ (Equation (1)). The larger the value f_r_, the rougher the rock surface.

(1) $$f_{r}=\frac{S_{a}}{{\pi r}^{2}}$$

where:

f_r_—roughness factor [-];
S_a_—actual surface determined by the scanner [cm^2^];
r—radius of the drill core sample [cm].

## 3. Results

### 3.1. Concrete Mortar Properties

#### 3.1.1. Mechanical Properties of the Concrete Mortar

Table 2 shows the results of the bulk density ρ of hardened concrete, the uniaxial compressive strength of cubes f_c,cube_ and the splitting tensile strength f_t,cyl_ of the lab-produced cylindrical specimens according to the formulations of Table 1. Additionally, the values of the identical formulations for dynamically compacted test specimens are presented.

Table 2 clearly shows that the hardened concrete’s characteristics of the manually compacted samples are significantly lower than in the case of the dynamical compaction. The static compaction, which is only performed manually, results in a lower compaction effect.

The situation is similar for the samples taken in situ. The results are shown in Table 3.

#### 3.1.2. Concrete Mortar Durability

**Figure 9:**
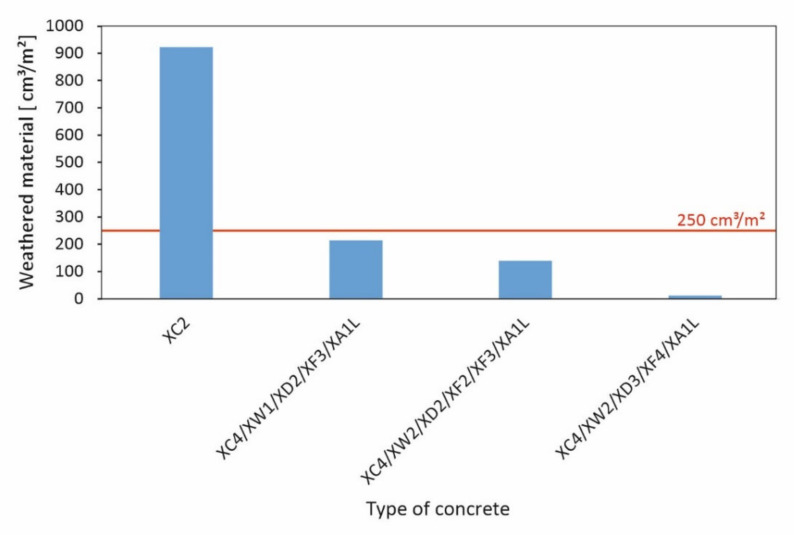
Results of the freeze–thaw tests according to [15] with 250 cm^3^/m^2^-criterion.

Each of the dynamically compacted specimens met the criterion for the frost resistance and the resistance to freeze–thaw with defrosting salt according to [15], meaning that the weathered material amount during the experiment was less than 250 cm^3^/m^2^. In the case of the statically compacted specimens, frost resistance could be verified for all tested concrete formulations; the frost-deicing salt resistance for all formulations except XC2, Figure 9. For the latter, the weathering was clearly too high, with a value of 923 cm^3^/m^2^.

**Figure 10:**
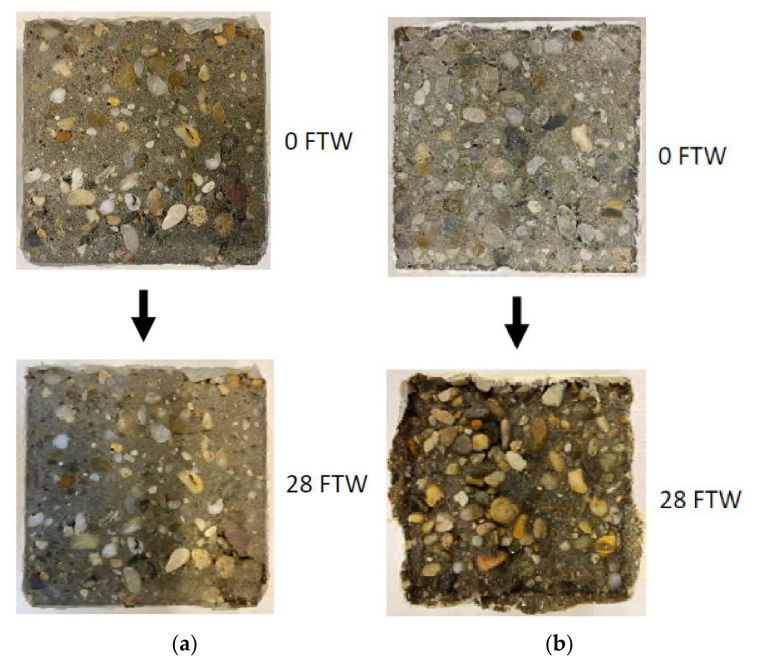
Weathering impact of 28 freeze–thaw cycles with defrosting salt: low weathering of the concrete XC4/XW2/XD3/XF4/XA1L (**a**), high weathering of the concrete XC2 (**b**); FTW = number of freeze–thaw cycles.

Figure 10 shows an example of exemplarily the weathering of the concrete class XC4/XW2/XD3/XF4/XA1L compared to concrete class XC2 compared after 28 freeze–thaw cycles as an example.

### 3.2. Contact Surface Identification

**Figure 11:**
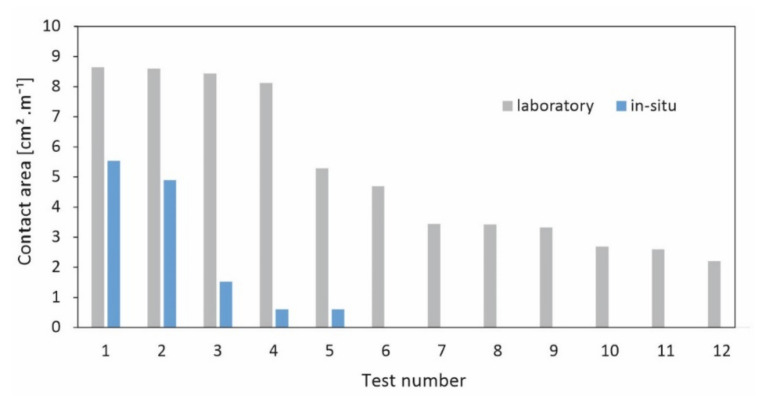
Results of the determination of the contact area between two stones in non-mortared constructions (considering an average stone mass of 1700–2100 kg).

The results of the tests carried out show that the contact area between the stones is extremely small in the range of a few cm^2^ and varies between 0.7 and 8.7 cm^2^ (Figure 11). The small contact area can cause considerably high compression stress, especially in the case of high stone walls due to high load, which in some cases exceeds the stone compressive strength. During the tests, it was recognized that this leads to a crushing of the stone at the pointed bearing areas, thus increasing the bearing surface and decreasing the compression stress.

### 3.3. Adhesive Tensile Strength and Shear Strength

**Figure 12:**
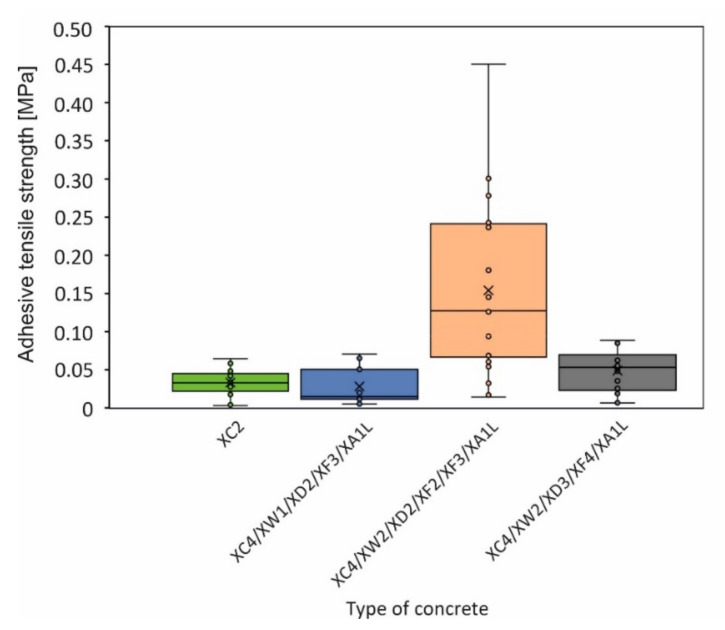
Boxplot of the adhesive tensile strengths between stone and concrete mortar for different concrete recipes.

**Figure 13:**
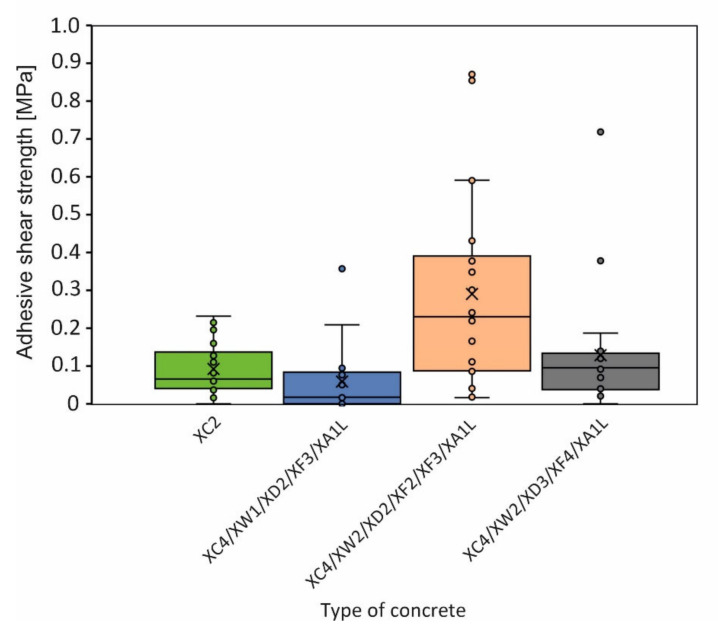
Boxplot of the adhesive shear strength between stone and concrete mortar for different concrete recipes.

The results of the adhesive tensile strength tests are shown in Figure 12; a clear relation to the concrete formulation is evident. The recipe XC4/XW2/XD2/XF2/XF3/XA1L shows the highest strength values as well as the largest scatter; the other concrete compositions are uniform. The adhesive shear strength shows partly similar characteristics (Figure 13).

**Figure 14:**
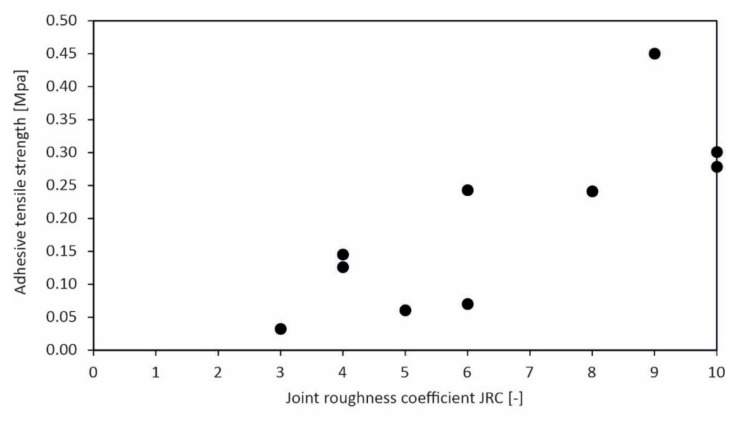
Comparing the adhesive tensile strength with the stone surface roughness JRC.

**Figure 15:**
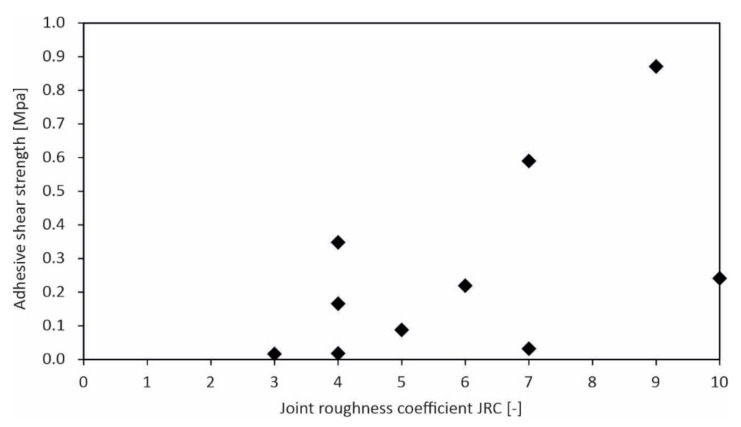
Comparison of the adhesive shear strength with the stone surface roughness JRC.

Comparing the strength values vs. the surface roughness of the stones, a certain correlation is noticeable, as exemplarily shown for the concrete formulation XC4/XW2/XD2/XF2/XF3/XA1L for both limestones (Figure 14). Strength shows an increasing trend with increasing roughness. The results of the adhesive shear strength indicate a larger scatter than the adhesive tensile strength in such a plot (Figure 15).

### 3.4. Roughness of the Stone Surface

**Figure 16:**
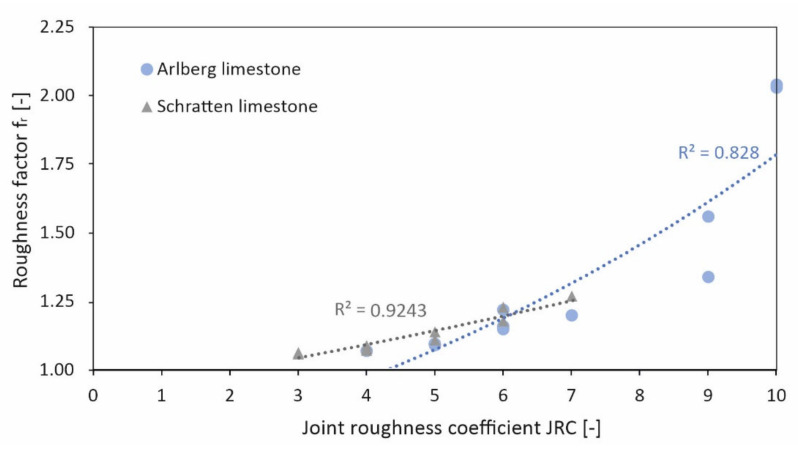
Comparison of the joint roughness coefficient JRC [16] with roughness factor f_r_ of two different types of limestone.

The considered limestone types have a roughness coefficient of JRC = 3 to 10. The roughness factor f_r_ determined from the comparison between the measured and theoretical surface shows values between f_r_ = 1.06 and 2.04. A clear correlation between roughness factor f_r_ and JRC is evident, while the Arlberg limestone shows a slightly higher roughness than the Schratten limestone (Figure 16).

### 3.5. Determination of the Shear Parameters

**Figure 17:**
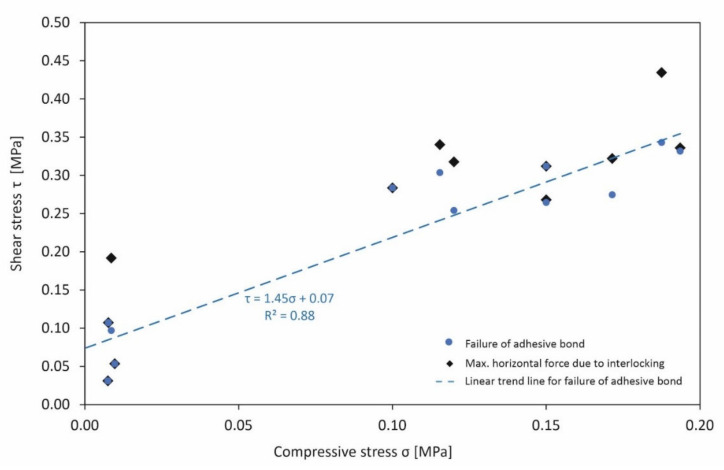
Determination of shear parameters and failure straight of a model stone structure.

Figure 17 shows the relationship between shear stress τ and compressive stress σ during failure at the bearing contact surface in accordance with the Mohr–Coulomb fracture criterion. A first maximum of the horizontal force was identified at the initial failure of the adhesive bond (“Failure of adhesive bond” in Figure 17), whereupon a further increase in force took place in most cases, depending on the morphological interlocking (“Max. horizontal force due to interlocking” in Figure 17).

The model tests show that there is a short load drop after the bond failure between the stone and the concrete mortar. The force then rises again due to the interlocking effect, because the impact of the stone roughness upon the friction angle usually significantly exceeds the initial bond stress (Figure 17). In addition to the individual results, Figure 17 also shows the empirical derivation of a regression for the cases of bond failure, where the coefficient of determination R^2^ = 0.88 is also given, which assumes a quite satisfactory value for this approach.

## 4. Discussion

Looking at the concrete strengths of the different mixtures and manufacturing methods (Table 2), it becomes clear that dynamic compaction results in a higher density of the concrete structure. Additionally, the mechanical properties of the dynamically compacted concretes are significantly higher than those of the statically compacted concretes, which comply with the in situ situation for the considered application. Insufficient static compaction results in significantly more pores, which have a negative effect on the concrete mortar strength. Accordingly, standardized fresh concrete that is ordered as ready-mixed concrete does not result in a standardized hardened concrete and does not necessarily meet the associated normative specifications because of inadequate compaction.

As far as the durability regarding frost and freeze–thaw resistance is concerned, the lower degree of compaction is disadvantageous only for mixture XC2; the other mixtures meet the requirements in terms of both frost and freeze–thaw resistance even in the statically compacted state.

Since the concrete quality (regarding cement content and w/b value) increases from XC2 to XC4/XW2/XD3/xF4/XA1L, at first glance, it is unusual that the two higher-grade formulations (XC4/XW2/XD2/XF2/XF3/XA1L and XC4/XW2/XD3/XF4/XA1L) do not necessarily have the highest compressive and splitting tensile strengths. This is since the two higher-grade formulations must have an air content of at least 2.5 and 4%, respectively, to meet the normative frost criterion, which is achieved by the addition of an air-entraining agent.

As far as the bond strength of the different concrete formulations to the rock surface is concerned, recipe XC4/XW2/XD2/XF2/XF3/XA1L stands out with significantly higher bond strengths. Accordingly, adhesion must be strongest for this mix design, although it has the lowest compressive strength and the second-lowest splitting tensile strength in comparison to the other recipes.

As expected, the identified roughness of the stones has a great influence on the bond strength between concrete mortar and rock due to increased interlocking and thus improved force transmission. The comparison of the visually determined roughness (via joint roughness coefficient JRC) and the roughness determined by laser scanning showed high conformity. Accordingly, the laser scanning method is also suitable for deriving the roughness of a rock surface. This method also gives the possibility to calculate a theoretical roughness factor to assign an exact roughness value to a specific rock surface; this approach can also be applied in the field.

The evaluation of the direct shear tests according to the approach of Mohr–Coulomb allowed the formulation of a trend line for the failure of the adhesive with a satisfactory coefficient of determination R^2^. The direct shear testing to determine the shear and compressive stresses showed that the roughness/unevenness of the fracture surface after the failure of the adhesive bond is also decisive for the shear forces to be transmitted due to interlocking.

The discussed shear relationship can be used as a basis that considers the failure of the cohesive bond. The interlocking that may occur subsequently after the failure of the cohesive bond can be concerned as additional safety regarding the failure of the structure accompanied by a significant deformation. This increasing deformation can be interpreted as a warning sign of failure, which can be seen as a safety criterion of supportive stone structures.

## 5. Conclusions

Supportive stone structures are standard safety measures that are widely used in road and railroad construction as well as in associated civil engineering. By mortaring the joint between the single stones, the combined masonry strength of the stone structure can be significantly increased. The different installation conditions of concrete mortar in this type of structure (compared to standard concrete that is dynamically compacted) result in properties that differ from those of conventional concrete, which has hardly been considered so far in research. In addition, the interaction between the concrete mortar and the stone for these structures has not yet been sufficiently investigated. This includes the recording of the surface condition and the mechanical interaction of rock and concrete mortar.

In the course of this work, the following conclusions could be drawn:

Regarding concrete mortar:

- The static compaction effect (by pressing the stones into the concrete mortar) does not achieve the same level of compaction as dynamic compaction, which results in a reduction in the bulk density and compressive strength of hardened concrete.
- The bulk density ρ of the statically compacted hardened concrete mortar is about 1 to 11% lower, the compressive strength f_c,cube_ is about 15 to 30% lower and the splitting tensile strength f_t,cyl_ is about 16 to 57% lower compared to dynamically compacted standard concrete.
- As far as the freeze–thaw resistance is concerned, statically compacted concrete shows high resistance to environmental influences: All mixtures met the requirements for frost resistance. Three out of four concrete mortar formulations achieved a sufficient freeze–thaw resistance, as demonstrated by a weathering volume of less than 250 cm^3^/m^2^.

Regarding bond strength between rock and concrete mortar:

- Different types of concrete mortar achieve different bond strengths—expressed as tensile strengths and shear strengths—when applied to a rock surface. Among these, the recipe XC4/XW2/XD2/XF2/XF3/XA1L shows by bar the highest strength values.
- The higher the roughness of the stone surface, the better the bond between the stone and the concrete mortar. An expected positive correlation between the roughness and the adhesive tensile strength can be demonstrated in the presented results, with the correlation to adhesive shear strength being less significant.
- It was possible to determine a failure description based on the Mohr–Coulomb fracture criterion, which can be used to determine characteristic parameters for the design of stone support bodies.

Further research in this area should consider (i) a recording of the concrete structure by means of thin sections in order to be able to record the concrete (micro)structure and the distribution of the air voids in detail, especially in concrete mortar to stone contact area; (ii) the investigation of different stone geometries with different roughness. By increasing the number of samples, it will be possible to verify the conclusions drawn in this paper and thus make a more precise statement. Further research in this area is recommended with different stone geometries and different surface roughness.

## Figures and Tables

**Table 1:** Concrete formulations for investigating the properties of fresh and hardened concrete mortar.

Exposure Classes Acc. [11]	Concrete Compressive Strength Class Acc. [12]	Maximum Grain Size [mm]	Consistency: Compactability Acc. [12]	Water-Binder-Ratio W/B [-]	Cement Content [kg/m^3^]	Hydraulically Active Additives [kg/m^3^]
XC2	C25/30	16 and 22	C1 and C2	0.55	257	26
XC4/XW1/XD2/XF3/ XA1L	C25/30	16	C1	0.52	275	33
XC4/XW2/XD2/XF2/ XF3/XA1L	C25/30	16	C1	0.46	321	30
XC4/XW2/XD3/XF4/ XA1L	C25/30	16	C1	0.42	378	33

**Table 2:** Hardened concrete mortar properties of the laboratory-produced specimens (mean value from 3 tests in each case).

Concrete Exposure Classes Acc. [12]	Bulk Density ρ [kg·m^−3^] Statically/Dyn. Compacted	Compressive Strength f_c,cube_ [MPa] Statically/Dyn. Compacted	Splitting Tensile Strength f_t,cyl_ [MPa] Statically/dyn. Compacted
XC2	2072/2339	25.4/32.6	1.0/2.3
XC4/XW1/XD2/XF3/XA1L	2246/2303	24.5/34.9	1.7/2.9
XC4/XW2/XD2/XF2/XF3/XA1L	2199/2226	21.9/27.9	1.5/2.7
XC4/XW2/XD3/XF4/XA1L	2136/2161	28.4/33.5	2.6/3.1

**Table 3:** Hardened concrete mortar properties of the in situ taken drill cores shown as minimum (min.) and maximum (max.) values, and as arithmetic mean (mean value of at least three tests in each case).

Concrete Exposure Classes Acc. [12]	Bulk Density ρ [kg·m^−3^] Min/Max/Mean	Compressive Strength f_c,cyl_ [MPa] Min/Max/Mean	Splitting Tensile Strength f_t,cyl_ [MPa] Min/Max/Mean
XC3/XD2/XF3/XA1L	1822/2299/2079	2.88/12.48/8.9	0.45/2.25/1.6

## Data Availability

All data used are within this contribution.
